# Surgery for total anomalous pulmonary venous connection and long term follow up

**DOI:** 10.1186/1749-8090-8-S1-O260

**Published:** 2013-09-11

**Authors:** Y Menaissy

**Affiliations:** 1 Cardiothoracic Surgery Department, Cairo University, Cairo, Egypt

## Background

We report a retrospective analysis of the demographic and clinical profiles of patients in order to assess the results of operative repair for total anomalous pulmonary venous connection (TAPVC) and the long term follow up.

## Methods

In the period between January 1998 and April 2013, 84 patients (48 boys) underwent repair for total anomalous pulmonary venous connection. Their ages ranged from 2 weeks to 1.6 years (mean 4 months). The patients’ weight ranged from 3 to 8 kg (mean 5 kg). About 71% of patients (n=60) were less than the 50th percentile of predicted weight for age and sex. The anomalous connection was supracardiac in 69 (82%), cardiac in 9 (11%), infracardiac in 3(3.6%) and mixed in 3(3.6%) patient. Eighteen (21%) patients had obstructed drainage and 27 patients (32%) had moderate or severe pulmonary arterial hypertension. Fifteen patients (18%) had to be operated upon on an emergency basis. For supracardiac and infracardiac connections, transcardiac approach was used for anastomosis. In cardiac type, coronary sinus was unroofed and the resultant defect along with atrial septal defect was closed with a single patch.

## Results

All the patients were operated upon using moderately hypothermic cardiopulmonary bypass except 4 patients (circulatory arrest was used). There were 4 (4.7%) in-hospital deaths. Three patients died of pulmonary arterial hypertensive crisis and one developed severe chest infection (died 3 weeks post-operatively with no pulmonary venous obstruction). Follow-up ranged from 6 to 181 months (mean 73 months). There were no late deaths, but 5 patients developed venous obstruction and required balloon dilatation.

## Conclusion

Mortality continues to be relatively high in infants with total anomalous pulmonary venous connection. Severe pulmonary arterial hypertension appears to be the most important predictor of operative mortality. Severe malnutrition, delayed diagnosis and late referrals possibly contribute to the high mortality.

